# Gastroscopy after positive screening for faecal immunochemical tests and colonoscopy: A systematic review

**DOI:** 10.1371/journal.pone.0281557

**Published:** 2023-02-10

**Authors:** Lina Choe, Jerrald Lau, Larry Teck-Seng Yip, Guowei Kim, Ker-Kan Tan

**Affiliations:** 1 Saw Swee Hock School of Public Health, National University of Singapore, Singapore, Singapore; 2 Yong Loo Lin School of Medicine, National University of Singapore, Singapore, Singapore; 3 University Surgical Cluster, National University Health System, Singapore, Singapore; 4 Division of Surgical Oncology, National University Cancer Institute, Singapore (NCIS), National University Health System, Singapore, Singapore; Changhua Christian Healthcare System: Changhua Christian Hospital, TAIWAN

## Abstract

**Background:**

Colorectal cancer (CRC) screening using the faecal immunochemical test (FIT) kits based on the detection of occult blood in stool is widely advocated in numerous screening programs worldwide. However, CRC is not commonly diagnosed in positive cases. We undertook this review to determine if there is evidence to suggest the use of opportunistic oesophago-gastro-duodenoscopy (OGD) in patients without CRC.

**Methods:**

A systematic review encompassing three electronic databases was performed. All peer-reviewed studies of FIT-positive patients who underwent either OGD and colonoscopy concurrently or OGD post-colonoscopy were included. Only studies from 2008 to 2022 using FIT kits were included to ensure studies not previously included in an earlier review were being analysed. A forward citation search of the included articles was also conducted to ensure no relevant articles were missed.

**Results:**

A total of 2409 records were extracted. Only four studies fulfilled the selection criteria and were included. Although the rates of abnormal OGD results were relatively high in the four studies, only 3 of 605 (0.50%) patients had gastric cancer in the entire review sample. No other malignancies were identified in all four studies. Other notable pathologies such as gastric polyps and gastritis were also reported.

**Conclusions:**

There is little overall evidence to recommend UGI screening for all FIT-positive patients following a colonoscopy. However, there may be a role for clinicians to consider opportunistic OGD in this group of patients. Future research should examine patient populations from other sociocultural contexts including cost-effective analysis when considering changes in health guidelines on UGI screening.

## Introduction

Colorectal cancer (CRC) is one of the top cancers globally, and screening has been shown to improve oncological outcomes [1]. In most CRC screening programs worldwide, faecal occult blood test (FOBT) remains the most frequently recommended screening modality. FOBT acts by detecting small amounts of blood in the faeces which could indicate bleeding in the intestinal tract [2]. A patient with a positive FOBT would be recommended to undergo colonoscopy, but the majority will test negative for CRC [3]. Thus, the ongoing question among clinicians is whether there is another bleeding source proximal to the colon that may account for the positive stool test instead [4].

Various studies including a systematic review have been carried out to determine the possibility of an upper gastrointestinal (UGI) source of bleeding following a negative colonoscopy in patients with a positive FOBT [5–7]. Unfortunately, the systematic review surmised that evidence to recommend screening of the UGI tract in FOBT-positive but colonoscopy-negative patients from the selected studies was inconclusive [7]. However, many of the studies in the previous review used guaiac-based kits (gFOBT) and were published numerous years back [7]. In the past two decades, countries have increasingly adopted the faecal immunochemical test (FIT) kit instead of gFOBT due to its superior sensitivity and specificity [8]. Due to the breakdown of haemoglobin in the lower GI tract, FIT kits should not be able to detect UGI bleeding, unlike gFOBT.

Thus, while the relevance of the aforementioned systematic review which focused on gFOBT is no longer reflective of the most current clinical practice, the question of whether to opportunistically conduct a UGI scope, or oesophago-gastro-duodenoscopy (OGD), in this subset of patients remains unanswered. We therefore undertook this systematic review to specifically identify and synthesise all studies investigating the association between patients with positive FIT results who have undergone colonoscopy and gastroscopy to better understand whether clinicians should consider an opportunistic OGD in FIT-positive/colonoscopy-negative patients.

## Materials and methods

### Study eligibility criteria

The present systematic review included all studies which involved (I) FIT-positive patients who underwent a colonoscopy (II) and went for UGI screening concurrently or post-colonoscopy, (III) were peer-reviewed with full texts in English, and (IV) were published between 2008–2022. Inclusion criteria IV was used to ensure that the current review would only examine studies published after the search period captured by Allard et al.’s prior systematic review [7]. Articles were excluded if they were (i) not peer-reviewed and (ii) articles that did not include primary data (e.g. reviews, meta-analyses, commentaries, editorials).

### Search strategy

Keywords, titles, and abstracts in three electronic databases (PubMed, CINAHL and Scopus) were searched using appropriate text words and related terms pertaining to colonoscopy, gastroscopy and gastrointestinal neoplasms. MeSH terms were utilised where possible. The search was conducted on articles published on these respective databases from 01 January 2008 through 09 June 2022. To improve the thoroughness of our search strategy, hand-searching of reference lists and a forward citation search from the included articles was also performed. The full search strategies for the respective databases can be found in S1 Appendix.

### Selection of studies

Following extraction of records from the search yields of each electronic database, EndNote X9 was utilised to remove duplicates. Titles and abstracts of each record were reviewed independently by two authors (LC and LT-SY) using the aforementioned inclusion and exclusion criteria. Thereafter, full-texts of all records that met the criteria were screened and discussed between the same two authors. A third author (JL) resolved any cases of disagreements. The reference lists from the included articles were hand-searched by LC and LT-SY to check for additional studies of relevance. After confirming the studies to be included, LC and LT-SY performed a quality appraisal on each of the studies included in the final sample using the appropriate Joanna Briggs Institute (JBI) critical appraisal tool, depending on the study design [9].

## Results

### Article evaluation

**Fig 1:**
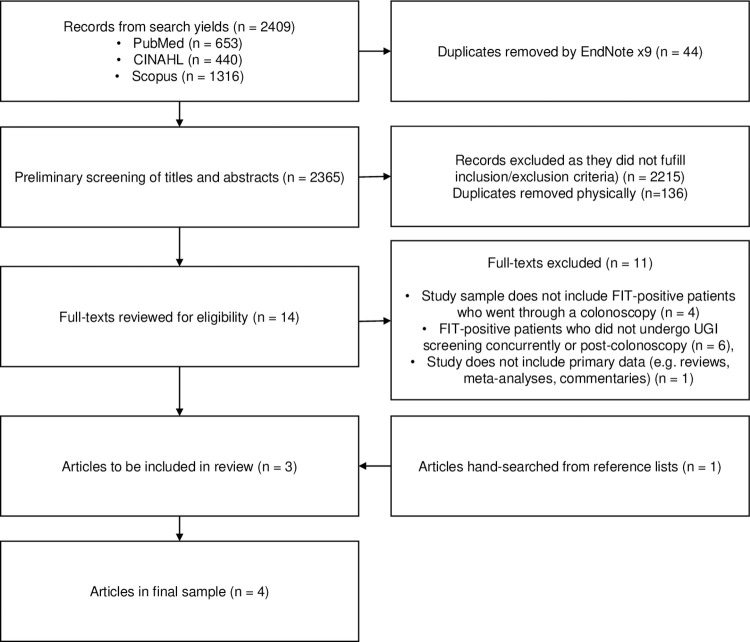
PRISMA flow diagram of study selection process.

A total of 2409 records were extracted from the three electronic databases. Forty-four duplicates were detected by EndNote, with an additional 136 manually detected and removed by the authors. The initial screening of titles and abstracts resulted in 14 articles that seemed to meet the criteria and subsequently underwent full-text review. Of these, only three articles (from PubMed and Scopus) fulfilled the selection criteria and were retained [10–12]. Unfortunately, CINAHL did not yield any papers of relevance. The fourth and final article included was from the forward citation search [13]. The detailed breakdown of the 2409 records can be seen in the flow diagram, **Fig 1**.

The JBI critical appraisal tools cohort study checklist was used for all four articles included [10–13]. Overall, all four studies had high quality, fulfilling more than 85% of the critical appraisal criteria–in which higher scores suggest more robustly designed studies with a lower likelihood of bias influencing the findings. Key characteristics from each included article (see Table 1) were then recorded by two authors (LC and LT-SY) using a standardised data collection form.

**Table 1:** Key characteristics, main findings, conclusions and limitations of the included studies.

Study	Krustri et al. (2018) [10]	Choi et al. (2013) [11]	Ng et al. (2016) [12]	Planade et al. (2021) [13]
**Study design**	Retrospective cohort	Retrospective cohort	Retrospective cohort	Retrospective cohort
**Country study was conducted**	Thailand	South Korea	Singapore	France
**Aims and objectives**	To determine if OGD should be performed in patients with positive FIT and negative colonoscopy results	To investigate the upper gastrointestinal (GI) condition confirmed by endoscopy in positive FIT patients without advanced colorectal neoplasia, haemorrhoid, or colitis	To evaluate the yield of OGD in patients with positive FITs	To evaluate the frequency of upper digestive lesions detected by upper endoscopy performed concomitantly with colonoscopy following a positive faecal immunochemical test
**Methodology**	Retrospective review of patient’s medical records	Retrospective review of patient’s medical records	Retrospective review of patient’s medical records	Retrospective review of patient’s medical records
**Data collection period**	1 January 2015–31 December 2016	January 2005 –October 2011	January 2008 –December 2012	May 2016 –May 2019
**Study inclusion criteria**	FIT positive patients who underwent colonoscopy and OGD on the same day with negative colonoscopy results	Patients who underwent both OGD and colonoscopy three months before or after positive FIT	Patients who underwent concurrent or subsequent OGDs for positive FIT patients	Patients who had concomitant OGD and colonoscopy performed after positive FIT
**Study exclusion criteria**	Active upper or lower gastrointestinal bleeding, pre-existing gastrointestinal disease and previous gastrointestinal tract surgery	Patients with sepsis, on oral iron supplements, or/and with incomplete endoscopy	NIL	Patients who have undergone an upper endoscopy in the past 10 years, has upper gastrointestinal symptoms or history,
**Study sample**	n = 2629	n = 2565	n = 202	n = 143
**Groups of patients**	Symptomatic– 66.88% Asymptomatic– 33.12%	Symptomatic– 21.13% Asymptomatic– 78.87%	Symptomatic– 64.80% Asymptomatic– 35.20%	Symptomatic– 30.07% Asymptomatic– 69.93%
**Symptoms of symptomatic patients**	Dyspepsia Bowel habit change Anemia Weight loss	Nil	Dyspepsia Anemia Weight loss Suspected gastrointestinal bleed Follow-up of previous peptic ulcer	Upper digestive symptoms PPI intake Anemia History of Head and Neck cancers Endoscopic monitoring Others
**Sub-sample of interest–FIT-positive patients who underwent both colonoscopy and OGD**	n = 185	n = 340	n = 202	n = 100
**Sub-sample of interest–FIT-positive/colonoscopy-negative patients who underwent OGD**	NIL	n = 243	NIL	n = 63
**Sub-sample of interest–abnormal OGD**	n = 160/185 (86.49%)	n = 58/243 (23.87%)	n = 106/202 (52.48%)	n = 39/63 (61.90%)
**Sub-sample of interest–gastric polyp (e.g. adenoma, fundic gland polyps, hyperplastic polyps)**	n = 15/185 (8.11%)	n = 5/243 (2.06%)	NIL	n = 2/63 (3.17%)
**Sub-sample of interest–gastric cancer**	NIL	n = 3/243 (1.23%)	NIL	NIL
**Main findings and conclusions**	OGD cannot be used to screen for gastric cancer among patients with positive FIT results but has a high yield in detecting benign conditions. As the cost of OGD in Thailand is inexpensive, dual endoscopy may be cost-effective to reduce the cost of treatment by reducing risk of gastric cancer especially in patients with *H*. *pylori*.	OGD/UGI evaluation should be additionally performed in patients with positive FIT and negative colonoscopy in Korea as the study has shown that incidence of gastric cancer in patients with positive FIT is higher than the general population in Korea.	It is not necessary for patients with FIT positive to go for routine gastroscopy from an oncological standpoint as no UGI cancers were detected in the study. Patients with FIT positive results should go for a routine gastroscopy as there is a high diagnostic yield for abnormalities in the UGI.	OGD may be effective in screening for upper digestive lesions in France due to the minimal added cost of OGD as compared to the cost of colonoscopy.
**JBI scoring**	87.5%	88.9%	87.5%	87.5%

### Study characteristics

All four studies reviewed patient’s medical records retrospectively to examine if OGD should be conducted in patients with positive FIT results [10–13]. Patients in all four studies had positive FIT results and underwent OGD concurrently with or after colonoscopy. Three of the four studies stratified their respective samples to include various patient sub-groups such as (i) FIT-positive/colonoscopy-negative patients, (ii) FIT-positive/colonoscopy-positive, (iii) FIT-positive/colonoscopy-negative/OGD-positive or (iv) FIT-positive/colonoscopy-negative/OGD-negative results [10, 11, 13]. Krustri et al., Choi et al. and Planade et al. managed to specifically study the OGD results of patients who had positive FIT *and* negative colonoscopy results [10, 11, 13]. Ng et al., however, were less specific and included all FIT positive patients who underwent concurrent OGD [12].

A full description of study characteristics such as the study design, country of study, aims and objectives, methodology, data collection period, key findings, and JBI scoring can be seen in Table 1.

### Outcomes of interest

#### Abnormal OGD results

Abnormal OGD results in the four articles largely referred to gastric cancer (advanced and early), gastritis/duodenitis (erosive or non-erosive), *Helicobacter pylori* infection, peptic ulcer disease, gastric ulcer, gastric polyp, hiatal hernia, duodenal ulcer, etc. Abnormal OGD results in Krustri et al., Choi et al. and Planade et al. were based on FIT-positive/colonoscopy-negative patients while Ng et al. covered all FIT-positive patients who underwent colonoscopy [10–13]. Abnormal OGD results were relatively prevalent, with three studies finding more than 50.00% of their sample with abnormal OGD findings [10, 12, 13].

#### Gastric polyp and cancer

The incidence of gastric cancer was extremely low. Only three of 605 (0.50%) patients in the total sample, all of which were found in Choi et al.’s study, had gastric cancer [11]. No other UGI cancers were identified. Prevalence of gastric polyps among the sample population in the three studies was low, with less than 10.00% in each study.

#### H. pylori infection

Unlike gastric cancer, the incidence of *H*. *pylori* infection was found to be higher. In one study, 23 of 185 (12.43%) patients who were FIT-positive/colonoscopy-negative were found to be *H*. *pylori*-positive [10]. Of these, 18 of 23 (78.26%) of *H*. *pylori*-positive patients had dyspepsia. The other 5 of 23 (21.74%) patients infected with *H*. *pylori* were asymptomatic. In the other three studies, *H*. *pylori*-positive rates were 43 of 340 (12.65%), 29 of 202 (14.36%) and 12 of 63 (19.05%) respectively [11–13].

#### Gastritis and intestinal metaplasia

Gastritis and intestinal metaplasia were common findings observed within the included studies, with some 117 of 160 (73.13%) of patients with abnormal OGD results in Krustri et al.’s study found to have gastritis [10]. Among FIT-positive/colonoscopy-negative patients, 131 of 243 (54.01%) were identified to have chronic superficial gastritis or atrophic gastritis while the sample in Ng et al.’s study found that 89 of 202 (44.10%) had gastritis/duodenitis [11, 12]. In contrast, Planade et al. found a lower rate of gastritis/esophagitis at 14 of 63 (22.22%) as compared to the other studies [13].

## Discussion

This systematic review aimed to provide a better understanding on whether there is a need to perform opportunistic OGD in FIT-positive patients with negative colonoscopy findings. While the low rate of gastric cancer observed in our included studies seems unsupportive of routine OGD screening for patients with a positive FIT, three studies concluded that OGD should nonetheless be considered in this setting [10, 11, 13]. One possible rationale is that prior research has demonstrated higher rates of gastric cancer in Korea relative to the rest of the world [14]. This is supported by the Global Cancer Statistics (GLOBOCAN) 2018, where the incidence of gastric cancer was the highest within East Asia, almost 1.8 times more than the second highest region—Eastern Europe—with the Republic of Korea topping the chart for country with the highest incidence [15]. The prevalence of gastric cancer in Korea was 15171 per 100,000 in 2018 [16]. Since 1999, Korea has had a national screening programme that includes biennial gastric cancer screening for individuals 40 years and older. Hence, it would be intuitive to advocate OGD amongst Korean patients who have a positive FIT who have yet to undergo a screening OGD as per the guidelines regardless of the colonoscopy findings, as it is in line with national healthcare policy and the infrastructure already facilitates such a conclusion. Nevertheless, none of the studies included performed cost-effective analyses or examined the incidence of gastric cancer in patients who were asymptomatic but going for routine screening gastroscopy. The affordability of OGD and presence of tertiary healthcare support are just two of the numerous factors that must be considered in the clinical decision-making process of recommending a potential invasive procedure to patients with a positive FIT.

Apart from the incidence of gastric cancer reported among the studies, the clinical significance of diagnosing gastritis with concomitant *H*. *pylori* infection remains debatable although it has been listed as a definite carcinogen by the World Health Organisation (WHO) since 1994 [17]. In our review, approximately 10% of the total sample had *H*. *pylori* infection. This is lower than expected, given that prevalence can be as high as 50–90% in certain countries [18]. Specific to gastric cancer prevention, there may be some benefit in searching for and eradicating *H*. *pylori* to reduce the incidence of gastric cancer [19], though the data remains controversial [20]. If *H*. *pylori* was a concern, there are several alternative cost-effective and non-invasive tests to diagnose the infection [21, 22].

Besides *H*. *pylori* infection, patients with atrophic gastritis and/or intestinal metaplasia have a higher risk for gastric cancer [23, 24]. However, the extent, type and severity of these two pre-malignant conditions significantly influence the risk [25, 26]. This has led to guidelines from Europe and North America recommending gastric mapping during OGD followed by no routine surveillance, or surveillance OGD at 1- to 3-yearly intervals, to monitor for progression and to intervene via endoscopic mucosal resection or endoscopic submucosal dissection to prevent development into advanced gastric cancer. In FIT-positive/colonoscopy-negative patients, this may represent an opportunity to identify and potentially risk stratify patients with gastric intestinal metaplasia or atrophy, especially in countries that do not have a high prevalence of gastric cancer [27].

### Limitations across the included studies

Despite the 14 years that have elapsed between Allard et al.’s review and the present research, our review found that the existing body of relevant evidence examining whether OGD should be considered for FIT-positive/colonoscopy patients remains scant and derived from retrospective study designs [10–13]. Three of the four studies had relatively small study samples which may demonstrate skewed baseline characteristics and outcomes due to chance bias, leading to potential limitations in generalisability of the study findings [10, 12, 13]. Furthermore, small samples tend to produce smaller effect sizes, which could be a reason for the extremely low incidence of gastric cancer found in the included studies. The retrospective nature of the included studies means that the possibility of selection biases also cannot be discounted, as treating physicians may have offered OGD to certain groups of patients with higher inherent risk of gastric pathologies, rather than all patients with a positive FIT. Additionally, three studies were conducted in Asian populations where the incidence of gastric cancer and *H*. *pylori* infection is higher than that in the West [28]. These findings should therefore be generalised to Western populations only with some caution. The absence of cost-effectiveness data also makes it more difficult to conclude whether OGD should generally be considered by clinicians for patients with positive FIT results, as relatively low procedure costs and risk of complications to the individual can still add up to a substantial financial burden on the healthcare system at large.

Lastly, our review found dissimilarities in how specific the respective studies were in evaluating patient characteristics. For example, Ng et al. did not stratify FIT-positive patients by their colonoscopy result, and both Ng et al. and Choi et al. did not report their OGD findings based on whether patients were symptomatic or asymptomatic [11, 12]. Future studies should attempt to understand if OGD outcomes are significantly different across these patient sub-groups as these may further inform risk stratification protocols.

Taking these limitations into consideration, future research should prospectively establish clinical outcomes and cost-effectiveness of performing OGD on FIT-positive individuals using larger, more representative patient cohorts. Confounding factors, such as anaemia, related medical prescriptions, and baseline clinical and sociodemographic characteristics should also be taken into consideration, as these may influence the likelihood of bleeding lesions, leading to a higher incidence of false-positive FIT. While randomised or cluster randomised controlled trial designs would be ideal in establishing the clinical efficacy of OGD on such patients, the lack thereof is perhaps understandable given the financial, logistical and ethical challenges associated with the present research question.

### Limitations of the current review

Our review utilised a multi-database search strategy, with the inclusion of MeSH terms where possible to broaden the likelihood of capturing all articles of relevance. However, it is possible that we may have missed some studies as we were only able to include full-texts that were available in English, especially from studies with findings that may be more locally relevant and whose authors may have published in local or regional journals. Nonetheless, as our search strategy exhaustively examined the body of literature in three of the largest academic databases relevant to the research question, the findings of this review are still representative of the limited evidence internationally available to clinicians and healthcare researchers on recommending OGD in this patient population.

## Conclusions

There is little overall evidence to currently recommend UGI screening for all FIT-positive patients following a colonoscopy. However, there may be a role for clinicians to consider opportunistic OGD in this group of patients, guided by operational considerations, risk assessment, and the patient’s personal and family medical history. In addition, due to the lack of generalisability of findings from the current four articles which are mostly from Asia, future studies conducted should examine patient populations from other sociocultural contexts [1]. Cost-effectiveness analysis will also be important for healthcare systems intending to reconsider health policy and guidelines on UGI screening.

## Supporting information

S1 Checklist PRISMA 2009 checklist. (PDF)

S1 Appendix Search strategy. (DOCX)
