# Supplementary material for: Gastroscopy after positive screening for faecal immunochemical tests and colonoscopy: A systematic review
Source: PLoS One. 2023 Feb 10;18(2):e0281557. doi: 10.1371/journal.pone.0281557 (PMC9916633; doi:10.1371/journal.pone.0281557)
Supplement: S1 Appendix — (DOCX) [file pone.0281557.s002.docx]

# Appendix I

Appendix I. Search strategy

PubMed (653 Searches)

(("Gastrointestinal Neoplasms" [MeSH])) AND (("FIT" OR "Fecal immunochemical test" OR "Faecal immunochemical test"))) AND (("Endoscopy, Gastrointestinal" [MeSH])) AND (English[Language]) AND 2008-2022

CINAHL (440 searches)

(("Gastrointestinal Neoplasms" [MeSH])) AND (("FIT" OR "Fecal immunochemical test" OR "Faecal immunochemical test"))) AND (("Endoscopy, Gastrointestinal" [MeSH])) AND (English[Language]) AND 2008-2022

Scopus (1316 searches)

( ALL ( ( "digestive system diseases" OR "gastrointestinal neoplasms" OR "gastrointestinal diseases" OR "stomach ulcer" OR "stomach neoplasms" OR "peptic ulcer" OR "peptic ulcer haemorrhage" OR "liver disease" ) ) AND ALL ( ( "fit" OR "fecal immunochemical test" OR "faecal immunochemical test" ) ) AND ALL ( ( "endoscopy" OR "colonoscopy" OR "esophagogastroduodenoscopy" OR "egd" OR "oesophago-gastro-duodenoscopy" OR "ogd" OR "gastroscopy" OR “capsule endoscopy” ) ) AND LANGUAGE ( english ) ) AND PUBYEAR > 2007
